# PMMA-based composite bone cements with zirconium oxide fillers of different granulations: structural optimization and biofunctional potential

**DOI:** 10.3389/fbioe.2026.1796706

**Published:** 2026-05-13

**Authors:** Robert Karpiński, Agata Przekora, Jakub Szabelski

**Affiliations:** 1 Department of Machine Design and Mechatronics, Faculty of Mechanical Engineering, Lublin University of Technology, Lublin, Poland; 2 Institute of Medical Sciences, The John Paul II Catholic University of Lublin, Lublin, Poland; 3 Department of Tissue Engineering and Regenerative Medicine, Medical University of Lublin, Lublin, Poland; 4 Faculty of Mechanical Engineering, Lublin University of Technology, Lublin, Poland

**Keywords:** bone cement, composite reinforcement, functional biomaterials, mechanical performance, particle size, PMMA, polymer-ceramic composite, zirconium oxide (ZrO2)

## Abstract

**Introduction:**

Polymethyl methacrylate (PMMA) bone cements are widely used in orthopaedics, but their limited fatigue resistance, brittleness and lack of biological bonding to bone motivate the search for composite formulations with improved mechanical performance and biofunctional potential. Zirconium oxide (ZrO_2_) is an attractive radiopaque ceramic filler that may reinforce the PMMA matrix while enabling microstructural optimisation.

**Methods:**

Commercial Refobacin Plus acrylic bone cement containing 0.6 g gentamicin was modified with ZrO_2_ particles of three granulations: nanoparticles (<100 nm), microparticles (≈5 μm) and fine powder (<10 μm), introduced at 1–5 wt%. After curing, specimens were subjected to compressive strength testing in accordance with ISO 5833, Shore D microhardness measurements and scanning electron microscopy to assess microstructure, particle dispersion and the occurrence of agglomerates or defects.

**Results:**

All PMMA/ZrO_2_ composites exhibited compressive strength above 70 MPa, thus meeting the ISO 5833 requirement. For ZrO_2_ contents up to about 3 wt%, compressive strength remained similar to the control cement; nanoparticle-filled samples maintained approximately 75–85 MPa, and fine and medium particles at 1–3 wt% often slightly increased average strength, whereas a 5 wt% admixture (especially of larger particles) significantly reduced compressive strength and hardness due to particle agglomeration. Hardness values were comparable to the unmodified cement at ≤3 wt% ZrO_2_ but decreased by roughly 18–21% at 5 wt%, and at a given concentration hardness was not markedly affected by particle size; SEM imaging revealed that homogeneously dispersed fine ZrO_2_ supported stress transfer and matrix stiffening, while large agglomerates acted as structural defects.

**Discussion:**

The results indicate that careful selection of ZrO_2_ grain size and limiting its content to low concentrations enables PMMA bone cement modification without compromising mechanical integrity, with ZrO_2_ functioning simultaneously as a reinforcing and radiopaque phase. Such tailored PMMA/ZrO_2_ composites may support the design of bone cements with optimised strength and biofunctionality, although further studies including fatigue tests and in vitro/in vivo biological evaluations are required to fully validate their clinical potential.

## 1 Introduction

Polymethyl methacrylate (PMMA)-based bone cements have been widely used in orthopaedics for over half a century as a material for fixing joint endoprostheses and filling bone defects (Karpiński and Szabelski, 2025; Kumar, 2025). Since their first clinical applications in the 1960s, they have become an indispensable element of many orthopaedic surgical procedures, such as joint replacement surgery and percutaneous stabilisation of vertebral fractures (vertebroplasty, kyphoplasty) (Enache et al., 2025; Kim et al., 2025; Mu et al., 2025). The popularity of PMMA cements is due, among other things, to their favourable performance characteristics and ease of preparation. The two-component mixture (powder + liquid) solidifies within a few minutes, ensuring the initial stabilisation of the implant in the bone within a very short time after application (Kumar and Ghosh, 2021; Ghasemi et al., 2023; Seesala et al., 2024). This technique of fixing implants or filling bone defects allows the operated limb to be loaded quickly, which speeds up rehabilitation and, as a result, the patient’s recovery (Liu et al., 2024; Wilczyński et al., 2024; Karpiński et al., 2025).

Despite their undoubted advantages, conventional PMMA bone cements have significant limitations that reduce their long-term clinical effectiveness. First and foremost, they are biologically inert materials; once hardened, the cement does not form a chemical or biological bond with the surrounding bone, which means that the implant does not undergo osseointegration (Gürses and Ejder-Korucu, 2016; Hussain and Al-Sarraf, 2022; Hong et al., 2025). A number of technological and environmental factors may also contribute to the deterioration of mechanical properties, such as disturbances in the proportions of components during preparation, as well as contamination of the cement structure with blood, bone fragments or saline solution during implantation (Karpiński et al., 2019b; 2019a). In addition, cement has relatively low fatigue strength and brittleness, which in the long term promotes the initiation of cracks and damage to the cement, which can lead to loosening of the implant and the need for surgical revision (Kuehn et al., 2005; Amerstorfer et al., 2017). Another problem is the highly exothermic polymerisation reaction: during the setting of PMMA cement, a significant amount of heat is released, which can cause damage (thermal necrosis) to the surrounding bone tissue (Szoradi et al., 2024; Tanvir et al., 2025). In addition, the liquid monomer remaining in the hardened cement may have a cytotoxic effect on cells. The PMMA surface is also susceptible to bacterial adhesion. Unmodified cement, as a biopassive material, does not inhibit microbial colonisation, which increases the risk of biofilm formation and periprosthetic infections. Therefore, cements supplemented with agents such as gentamicin, tobramycin, vancomycin or, for example, amphotericin are often used to give the cements fungicidal and bactericidal properties (Kwong et al., 2024; Buchholz et al., 2025; Frank et al., 2025; Humez et al., 2025). In view of the above, work has been underway for many years on modifying the composition of bone cements in order to increase their mechanical strength, improve their bioactivity and reduce the negative thermal and biological effects of polymerisation. One promising strategy is to introduce solid phases in the form of inorganic particles (especially ceramics) into the PMMA matrix, which can simultaneously perform reinforcing and bioactive functions. Thanks to such additives, the cement could form a more durable bond with the bone and be more resistant to fatigue cracking, as well as hinder bacterial colonisation without the need for antibiotics.

To date, many different additives have been tested in PMMA cements to improve their properties. Particular attention has been paid to bioactive ceramic materials, such as hydroxyapatite (HA) (Karpiński et al., 2024b; Kırkbınar et al., 2024; Zhou et al., 2024; Krishnan et al., 2025b), tricalcium phosphate (TCP) (Santos et al., 2019; Constant et al., 2022; Hossain et al., 2023; Karpiński et al., 2024a; Cen et al., 2025) and bioactive glass, which, when added to cement, give it osteoconductivity and the ability to bond with natural bone tissue. Another approach is to reinforce the cement by adding high-strength fibres or particles, including carbon, glass or polymer fibres, as well as nanoparticles and nanofibres (e.g., carbon-based), which improve fracture resistance and can modify the cement setting process (Karpiński et al., 2024c). A standard component of most clinical bone cements is also radiopaque powder, added to provide contrast on X-ray images. Most often barium sulphate (BaSO_4_) at 10–15 wt% is used (Mashak et al., 2025). However, the presence of such foreign particles in the acrylic matrix has its price: it is believed that BaSO_4_ particles can act as crack nuclei, reducing the fatigue resistance of the hardened cement. Therefore, alternative ceramic fillers with better mechanical and biological compatibility are being sought. The literature has explored, among other things, the replacement of BaSO_4_ with other radiopacifiers, such as titanium (IV) oxide (TiO_2_), zirconium (IV) oxide (ZrO_2_) and others, in the hope of improving the fatigue strength and biological properties of the cement (Sari et al., 2024; Xu et al., 2024; Krishnan et al., 2025a).

However, research on PMMA-ZrO_2_ composites to date is limited, and the results obtained are not always conclusive. For example, Nishio Ayre et al. conducted analyses in which BaSO_4_ was replaced with ZrO_2_ (stabilised Y_2_O_3_) or TiO_2_ particles in typical clinical cement and found that the addition of >10% by weight of these radiopacifiers reduced the flexural strength and fracture resistance of the cement due to particle agglomeration and the formation of microvoids (Ayre et al., 2021). These results suggest that an excessive amount of added ceramic particles may act as inclusions that weaken the cohesion of the material, unless they are properly dispersed in the matrix. In turn, Chen and colleagues used nanometric ZrO_2_ as a carrier for antibacterial agents and obtained a cement with a strong effect against Gram-positive (*Staphylococcus aureus*) and Gram-negative (*Pseudomonas aeruginosa*) bacteria (Chen et al., 2022). Importantly, this modification, with a ZrO_2_ nanoparticle content of approximately 7% by weight, did not impair the mechanical properties of the material; the modified cement retained its strength in the three-point bending test, comparable to commercial cement, and even showed a slightly higher modulus of elasticity (Chen et al., 2022). Other studies report an increase in cytocompatibility (osteoblast density) after the addition of ZrO_2_ particles, especially in nano and functionalised form. For example, Gillani et al. reported increased bone cell (osteoblast) colonisation density on the cement surface after 24 h in the case of cement containing ZrO_2_ nanoparticles compared to both unmodified cement and cement with conventional BaSO_4_ (Gillani et al., 2010). However, this does not provide grounds for concluding that the addition of zirconium oxide alone made the cement bioactive in the classical sense (formation of an apatite layer, direct bond with bone).

The above results prove that the addition of zirconium can bring measurable benefits, but at the same time reveal certain limitations (e.g., a decrease in strength at excessive filler content or poor dispersion). However, there is a lack of comprehensive studies that would simultaneously analyse the influence of ZrO_2_ grain size and its content in cement on the full set of material properties - mechanical, thermal and biological. Previous analyses have only covered strength in a three-point bending test, which, although an important indicator of mechanical resistance, does not fully reflect the actual working conditions of cement in the human body. In practice, this material is primarily subjected to compressive stresses, so it would be equally important to examine its behaviour in compression tests. In other words, the optimal structural parameters (granulation and concentration) of zirconium filler that would allow for the production of cement with improved performance without negative side effects have not yet been determined. This research gap hinders the full exploitation of the potential of this additive in practice and indicates the need for further systematic research in this area.

The aim of this study was therefore to supplement the current state of knowledge by examining PMMA composite bone cements with zirconium oxide admixtures of varying particle sizes and concentrations. Samples of cement cured under standard conditions were evaluated for their physicochemical and mechanical properties (with particular emphasis on microstructure, compressive strength and hardness tests). The tests carried out made it possible to estimate the optimal ranges of ZrO_2_ content and particle size, allowing for a favourable modification of the properties of PMMA cement without compromising its structural integrity.

This study contributes to addressing an existing gap in the literature by providing a systematic evaluation of the combined effects of zirconium oxide particle size and concentration on the compressive behavior, microstructure, and hardness of PMMA bone cements. In particular, it extends previous studies by considering clinically relevant compressive loading conditions and identifying ranges of parameters that allow material modification without compromising structural integrity.

The PMMA/ZrO_2_ composite bone cements developed in this work are in line with the current direction of development of medical biomaterials, in which structurally optimised polymer-ceramic composite systems are replacing traditional single-phase materials. This study is of significant importance in light of the growing demand for composites with increased durability and biofunctionality, and the approach proposed in this work, consisting of simultaneous analysis of grain size and concentration in the context of mechanics, microstructure and ISO standard restrictions, is an innovative and comprehensive model for the evaluation of medical composites intended for orthopaedic applications. This study advances the field by providing a systematic and multi-scale evaluation of the combined effects of ZrO_2_ particle size and concentration on compressive strength, hardness, and microstructure of PMMA-based bone cements, which has not been comprehensively addressed in prior studies. Unlike previous works focused mainly on flexural properties or single filler characteristics, this research identifies optimal reinforcement ranges under clinically relevant compressive loading conditions, offering practical guidelines for structural optimization of bone cements.

## 2 Materials and methods

### 2.1 Material and sample preparation

In this study, composite bone cements were prepared by modifying commercially available medium-viscosity acrylic cement Refobacin® Plus Bone Cement (Zimmer Biomet), containing 0.6 g of gentamicin per portion. This cement is a widely used clinical formulation intended for anchoring joint endoprostheses, revision arthroplasty procedures and filling structural bone defects. Like typical PMMA cements, it comes in a two-component form: the liquid phase contains MMA monomer (with N,N-dimethyl-p-toluidine, hydroquinone and dye), while the powder phase consists of PMMA polymer, radiopaque agent (ZrO_2_), peroxide initiator and antibiotic. Its widespread clinical use, well-known setting kinetics and mechanical stability make this cement a suitable reference material for assessing the effect of reinforcing ceramics.

PMMA is responsible for forming a continuous structural phase that ensures load-bearing capacity and implant stability; however, this material is biologically inert and does not exhibit osteointegration properties. The ZrO_2_ additive serves as a radiopaque agent, allowing for the control of cement placement in X-ray imaging, and at the same time, thanks to its high hardness and chemical stability, it can improve the stiffness and resistance to microcrack initiation in the composite. The initiator and activator determine the course of the exothermic *in situ* polymerization reaction, whose parameters (setting time, maximum temperature) are of critical clinical importance. The presence of an antibiotic, such as gentamicin, provides local antibacterial activity and reduces the risk of peri-prosthetic infections without significantly affecting mechanical properties at controlled concentrations.

Zirconium dioxide (ZrO_2_) was selected as the reinforcing phase due to its unique physicochemical properties relevant to PMMA composites. ZrO_2_ is a biocompatible ceramic with high compressive strength, high hardness, fracture resistance, excellent chemical stability and natural high radiological density. The literature emphasises its high biocompatibility and lack of reactivity in a physiological environment, which ensures clinical safety. In addition, ZrO_2_ can improve the modulus of elasticity, microcrack initiation resistance and wear of polymer composites. Zirconium dioxide can also act as local nucleation centres during polymerisation, affecting heat distribution and potentially lowering the peak temperature of the exothermic cross-linking process. Therefore, ZrO_2_ is a promising additive for improving the structural properties of PMMA without compromising the integrity of the polymer matrix at an appropriately selected concentration.

Three types of zirconium dioxide powder, differing in particle size, were purchased for the study. A monoclinic ZrO_2_ powder with particle size <10 μm and purity 99.6% (Sigma-Aldrich, cat. No. 918849), a ZrO_2_ powder with particle size 5 μm, 99% trace metals basis (Sigma-Aldrich, cat. No. 230693), and a ZrO_2_ nanopowder with particle size <100 nm (TEM) and specific surface area ≥25 m^2^/g (Sigma-Aldrich, cat. No. 544760) were employed. The selection of these granulations enabled the analysis of scale-dependent strengthening effects resulting from different mechanisms of interaction with the PMMA matrix. Nanoparticles (<100 nm) are characterised by a very large specific surface area, which promotes strong interfacial interactions and improves composite packing. They can increase stiffness and resistance to microcrack initiation, but due to their tendency to agglomerate, they require evaluation of a safe concentration range. Microparticles ∼5 µm are the typical range used in polymer reinforcements. Their size promotes uniform dispersion and mechanical stability, allowing for potential crack bridging without significantly affecting viscosity and the polymerisation process. Particles <10 µm are an intermediate granulation variant, allowing for the evaluation of effects resulting from moderate specific surface area, impact on internal porosity, and effectiveness of interaction at the PMMA-ceramic phase boundary. The use of three different particle sizes allows the size with the most favourable reinforcement profile to be identified while maintaining stable process parameters and a homogeneous microstructure.

The base cement was tested in an unmodified (control) version and with the addition of ZrO_2_ in concentrations of 1%, 2%, 3% and 5% by weight (relative to the total weight of the cement). Zirconia powders were used as received from the manufacturers, without any additional treatment or pre-modification. Each ZrO_2_ fraction was first dry-mixed manually with the commercial cement powder in a clean glass beaker for approximately 30 s at room temperature, using a spatula to ensure thorough blending. The zirconia powders were taken from freshly opened containers and were free-flowing, which facilitated uniform distribution within the PMMA powder. They did not exhibit noticeable caking or lump formation during mixing. After this dry pre-mixing step, the powder blend was combined with the liquid monomer phase according to the manufacturer’s instructions, and mixing was continued until a homogeneous dough-like consistency was obtained.

### 2.2 Compressive strength tests

Compressive strength tests were performed to determine the effect of ZrO_2_ additives with varying particle sizes and concentrations on the mechanical behaviour of PMMA composite cements. The tests were performed in accordance with the recommendations of ISO 5833:2002 ‘Implants for surgery—Acrylic resin cements’, which is the reference standard for evaluating the performance of cements used in orthopaedics.

Cylindrical samples with dimensions of 6 ± 0.1 mm in diameter and 12 ± 0.1 mm in height were placed in an MTS Servohydraulic Bionix® Tabletop Test System, capable of precisely recording changes in force and displacement during loading. The samples were positioned axially, without spacers, and the parallelism of the end faces was checked to prevent uneven stress distribution during compression.

The load was applied continuously at a rate of 20 mm/min, in accordance with ISO guidelines. During the test, the system recorded the force-displacement curve, allowing for analysis of both the maximum values and the nature of the deformation until the sample was destroyed or reached its yield point.

The compressive strength value was taken as the maximum recorded force causing:

- Destruction of the sample (cylinder fracture),
- Or reaching a 2% permanent deformation (so-called 2% offset),
- Or exceeding the yield strength, whichever occurred first.

The force obtained was then divided by the initial cross-sectional area of the sample, expressing the result in MPa.

In addition, Young’s modulus, which is a measure of the stiffness of the material under compressive loads, was determined from the initial linear section of the force-deformation curve. This parameter allows the assessment of the effect of ZrO_2_ addition on the cement’s resistance to elastic deformation and, thus, on its potential behaviour after implantation when the cement is subjected to typical compressive loading.

Each measurement series was performed in accordance with the ISO standard on at least five samples, although in practice seven to eight samples were most often analysed, which increased the reliability of the average values and allowed samples with structural defects (voids, dispersion heterogeneity) to be rejected.

Compressive strength testing is a key step in the evaluation of PMMA composite cements, as compressive loading dominates in clinical conditions after endoprosthesis implantation. According to the literature and previous studies, slight changes in the microstructure of the cement (resulting from the mixing method, particle granulation or imperfect packing) can lead to significant differences in strength. Therefore, a detailed analysis of the nature of the damage and the variability of results in series is necessary to fully understand the effect of ZrO_2_ on the properties of PMMA.

### 2.3 Hardness testing

Hardness tests were performed to evaluate the resistance of PMMA/ZrO_2_ composite cements to local surface deformation, which may play an important role in the long-term mechanical stability of the cement after implantation. The Shore D hardness scale, commonly used in the analysis of polymeric materials, is particularly useful for evaluating materials with moderate elasticity, such as acrylic cements.

The samples for hardness testing were prepared in parallel with the compression samples, using the same batch of cement and the same mixing parameters to avoid differences resulting from the preparation process. Flat samples with a thickness of ≥4 mm were used for the measurements, in accordance with ISO 868:2003 ‘Plastics and ebonite—Determination of indentation hardness by means of a durometer (Shore hardness)’. Care was taken to ensure that the surface of each sample was smooth and uniform, free from visible air bubbles, and free from microdefects resulting from mechanical processing.

Hardness tests were performed using the Shore D scale in accordance with ISO 868:2003, using a stationary AFFRI® durometer. Flat samples with a thickness of ≥4 mm were placed on the base of the device and the indenter was applied perpendicular to their surface. After the reading stabilised (approx. 2–3 s), the hardness value was read. A minimum of three measurements were taken for each sample, and the arithmetic mean was taken as the final result. The measurement points were located at least 9 mm from the edge to avoid edge errors.

Shore D hardness complements strength analysis by reflecting the material’s resistance to local surface deformation. This parameter allows for the assessment of the homogeneity of ZrO_2_ particle dispersion, the degree of PMMA cross-linking, and the presence of microdefects or pores. Hardness testing also allows for the identification of possible changes in the behaviour of the composite surface, which is important for the long-term stability of the cement under endoprosthesis operating conditions.

### 2.4 Scanning electron microscopy imaging

Microstructural analysis of PMMA/ZrO_2_ composite cements was performed using scanning electron microscopy (SEM) to assess the effect of ZrO_2_ additive on the homogeneity of the PMMA matrix. The SEM imaging was also carried out to check whether the addition of ZrO_2_ particles negatively affects microstructural properties of the material, causing the formation of microstructural defects such as cracks and pores. This method is a standard tool in acrylic cement research, enabling detailed observation of the composite surface and analysis of the effect of both the concentration and granulation of the ceramic additive on the material’s microstructure.

For the SEM imaging, intact disk samples measuring 5 mm in diameter and 2 mm thick were prepared. They were taken from the same batch of cement used for the mechanical tests. The samples were cleaned, dried, and sputtered under a high vacuum with a 20 nm gold layer. The SEM (JEOL JCM-6000PLus, Tokyo, Japan) visualization was carried out in a high vacuum environment at low accelerating voltage (5 kV), allowing for a clear representation of the composite surface. For each sample, a series of images was captured at two magnification levels: ×200 and 500×, enabling the observation of both the macroscopic arrangement of composite components and local effect of the ZrO_2_ additive on material’s microstructure.

SEM analysis allowed us to assess the degree of integration of ZrO_2_ particles into the polymer matrix, the presence of potential agglomerates, and identify voids, microcracks, and other microstructural discontinuities that could affect the mechanical properties of the tested composites.

### 2.5 Statistical analysis

Statistical analysis was performed to assess the significance of differences between the results obtained for ZrO_2_-modified PMMA cements with different concentrations and particle sizes. The calculations were performed using the TIBCO Statistica 13.3 software package. Prior to the actual analysis, the normality of the data distribution was assessed using the Shapiro-Wilk test and the homogeneity of variance was verified using Levene’s test, which confirmed the applicability of parametric methods. Once these conditions were met, Tukey’s honest significant difference (HSD) *post hoc* test was used for comparisons between groups, which allows the identification of significant differences between multiple series of similar size. The significance level was set at α = 0.05. The results of mechanical tests, including compressive strength, Young’s modulus and Shore D hardness, were presented as mean values with standard deviation and then assigned to homogeneous statistical groups, which allowed the identification of ZrO_2_ concentrations and granulations causing changes that were significant from a mechanical and functional point of view. The statistical analysis enabled a full assessment of the influence of both particle size and concentration on the properties of PMMA composite cement.

## 3 Results

### 3.1 Compressive strength

**FIGURE 1:**
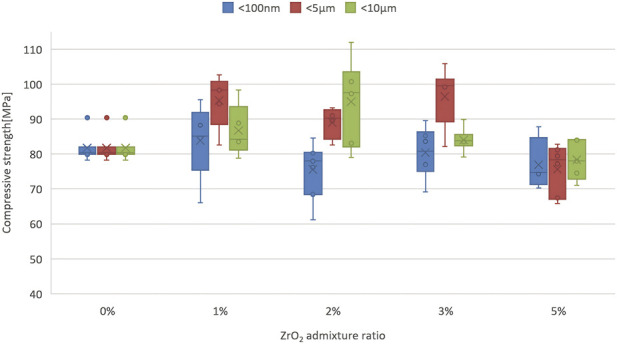
Compressive strength (mean ± SD, MPa; n = 7–8) of ZrO_2_-modified PMMA cements (0–5 wt%) with particle sizes 100 nm (nanoparticles), 5 μm (microparticles), 10 μm (fine powder). Error bars: standard deviation.

Figure 1 presents a summary of the maximum stress results for standardised bone cement samples with varying amounts of ZrO_2_ admixture in their composition and a reference sample–unmodified. The results obtained were characterised by small variations within individual series. The COV coefficient (defined as the ratio of the standard deviation of the series to the arithmetic mean) reached a maximum of 13% (average approx. 8%), which indicates that the cement samples were prepared correctly and consistently and that the strength tests were carried out correctly. The data obtained are homogeneous and not very scattered around the mean.

All mean results obtained for the tested bone cements, up to a maximum ZrO_2_ admixture of 5%, meet the minimum compressive strength criterion of greater than 70 MPa, as specified in ISO 5833. The only clearly visible reduction in strength can be observed with a 5% addition of ZrO_2_ with coarser granulations (5 μm and <10 μm). The finest admixture material in the tested admixture range does not seem to affect the compressive strength of cement; the values are relatively stable between 75 and 85 MPa, regardless of the degree of admixture. On the other hand, coarser powder increased the average compressive strength to some extent at a minimum amount in the cement composition (1%–3%), which seems to be consistent with prior studies that have shown that often a small amount of additives or even impurities had a positive effect on the strength of cement.

When analysing the impact of the granulation size of the admixture material, it should be noted that the differences in strength between individual series are insignificant and it is difficult to identify any universal patterns here. Only statistical analysis allows for a precise answer to the question of strength variability (Table 1) in the range of lower concentrations (1%, 2%, 3%). It has been confirmed that a significant reduction in the compressive strength of cement is observed when the additive level is too high. For the finest fraction (100 nm), no statistically significant differences between the averages were found. The safe modification range is therefore concentrations up to 3% — above this level, for larger particles, there is a clear negative effect documented statistically.

**TABLE 1:** Tukey’s HSD *post hoc* test for compressive strength (mean ± SD, MPa; n = 7–8). Values: p < 0.05; homogeneous groups indicated.

<100 nm					
Ratio	0% 82.161	1% 82.260	2% 74.663	3% 78.209	5% 77.660
0%	​	1.000	0.232	0.858	0.874
1%	1.000	​	0.336	0.846	0.865
2%	0.232	0.336	​	0.899	0.968
3%	0.858	0.846	0.899	​	1.000
5%	0.874	0.865	0.968	1.000	​
5 μm					
ratio	0% 82.161	1% 88.103	2% 85.801	3% 93.343	5% 74.823
0%	​	0.693	0.930	0.090	0.504
1%	0.693	​	0.986	0.781	0.048
2%	0.930	0.986	​	0.477	0.140
3%	0.090	0.781	0.477	​	0.003
5%	0.504	0.048	0.140	0.003	​
<10 μm					
ratio	0% 82.161	1% 82.176	2% 92.215	3% 82.928	5% 74.426
0%	​	1.000	0.187	1.000	0.429
1%	1.000	​	0.188	1.000	0.427
2%	0.187	0.188	​	0.253	0.003
3%	1.000	1.000	0.253	​	0.335
5%	0.429	0.427	0.003	0.335	​

In contrast, a counter-analysis, i.e., grouping fixed amounts of admixtures but with different granulation, allows for a comparison of differences between individual materials. Thus, groups of results that do not differ significantly in statistical terms were identified. In most cases (1% and 5%), both the finest particles (<100 nm) and larger fractions (5 μm, <10 μm) form a single statistical group, i.e., there are no significant differences between the average compressive strength values of cements with the same amount of different additives. This means that in these ranges of ZrO_2_ content, the granulation of the additive has no significant effect on the mechanical properties of the cement. Regardless of the selected fraction, the strengths obtained are comparable. The situation changes at concentrations of 2% and 3% (Table 2). The results are then clearly divided into two statistically homogeneous groups:

- Additive <100 nm, which in both cases shows lower average compressive strength,
- Larger fractions: 5 μm and <10 μm, with significantly higher compressive strength.

**TABLE 2:** Homogeneous groups (Tukey HSD, p < 0.05) for compressive strength (MPa) at 2 and 3 wt% ZrO_2_. Means ± SD; different numbers (1, 2) differ significantly.

​	2% ZrO_2_			3% ZrO_2_		
	Mean compressive strength [MPa]	1	2	Mean compressive strength [MPa]	1	2
<100 nm	74.66	****	​	78.21	****	​
5 µm	85.80	****	****	93.34	​	****
<10 µm	92.21	​	****	82.93	****	****

This separation indicates that at 2% and 3% addition, granulation becomes a decisive factor in the effectiveness of cement reinforcement, as the use of larger particles (5 μm, <10 μm) results in significantly better mechanical parameters than in the case of <100 nm admixtures.

### 3.2 Material elasticity

**FIGURE 2:**
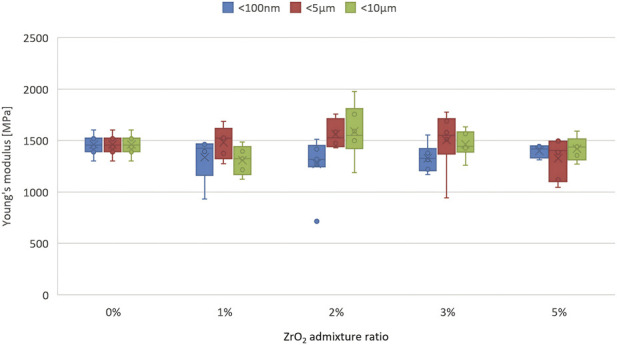
Young’s modulus (mean ± SD, MPa) vs. ZrO_2_ content (wt%) and particle size. Error bars represent SD from compression tests (n ≥ 5).

The analysis of Young’s modulus (Figure 2) depending on the amount of additive also does not give clear results, although the nature of the variability coincides with the obtained compressive strength results. The highest values are observed for thicker fractions of 5 µm and <10 μm at 2% and 3% additive, where the modulus reaches approximately 1,550 MPa. At the same time, the lowest values occur for 5% additive for both 5 µm and <10 µm (approximately 1,200–1,250). The fine fraction (<100 nm) shows less variability - its values are similar and range from 1,200 to 1,400, regardless of concentration.

The consistent change in both parameters and the fact that the changes are proportional may indicate that modification with the tested additive affects the entire microstructure of the material in an integrated manner, i.e., it does not introduce defects, weak points or dispersion of properties. This corresponds to both high-quality bonding and uniform dispersion of the additive, as a result of which no zones with weakened mechanical properties are formed in the tested material. The decrease in stiffness itself then leads to a decrease in compressive strength, which is typical for materials with low porosity and a well-bonded reinforcing phase (e.g., composites, ceramics).

When analysing constant amounts of admixtures with different granulation, it can be observed that for each additive level (1%, 2%, 3%, 5%), the highest values are most often observed for the 5 µm and <10 µm fractions. A particularly marked increase in values is visible for 2% and 3% - the values of the 5 µm and <10 µm fractions reach approx. 1,450–1,550 MPa, while for ZrO_2_ with a grain size of <100 nm they are significantly lower. For the highest additive concentration (5%), all fractions have lower values, below 1,350 MPa, which is consistent with previous observations - too high an amount of additive reduces elastic stiffness, which means greater susceptibility to deformation under the influence of forces. For the lowest concentration (1%) and the finest fraction (<100 nm), the results are the most stable (smallest error bars), but lower than for the optimal proportions of larger particles. It can therefore be seen that the best mechanical properties are obtained for medium (5 µm) and large (10 µm) granulation at a moderate additive concentration (2%–3%). Too much additive leads to a reduction in strength, while the finest particles ensure stability but not maximum parameters. It therefore seems that the optimal combination is larger particles and a moderate degree of admixture.

However, the results of statistical analysis show that in most cases there is no statistically significant difference between the individual mean values, with the exception of two cases: cement modified with the finest zirconium oxide (<100 nm) at a concentration of 2% compared to unmodified cement. Unmodified cement is not included in the homogeneous group of results No. 1, while cement modified with 2% zirconium oxide is not included in the homogeneous group of results No. 2 (Table 3).

**TABLE 3:** Homogeneous groups for Young’s modulus (mean ± SD, MPa; n ≥ 5) at varying ZrO_2_ (100 nm).

Ratio of <100 nm ZrO_2_	Mean Young’s modulus [MPa]	1	2
0%	1,492.717	​	****
1%	1,307.522	****	****
2%	1,165.144	****	​
3%	1,318.380	****	****
5%	1,264.137	****	****

Unfortunately, homogeneous groups overlap, and cements with different admixture compositions are included in both groups. It is therefore difficult to speak of the permanent nature of changes in the modulus in the range of 0%–5% on this basis. This may mean that the variability of the modulus is chaotic or that the fluctuations are within the measurement error limit. Perhaps the change in the modulus after an increase in the amount of additive from 2% to 3% and 5% means a gradual stabilisation of the modulus around a certain value (approx. 1,260–1,320 MPa). This would suggest that the maximum effect of the nanometric ZrO_2_ additive on the change in Young’s modulus occurs at 2%, and a further increase in concentration does not bring about statistically significant changes.

### 3.3 Surface hardness

**FIGURE 3:**
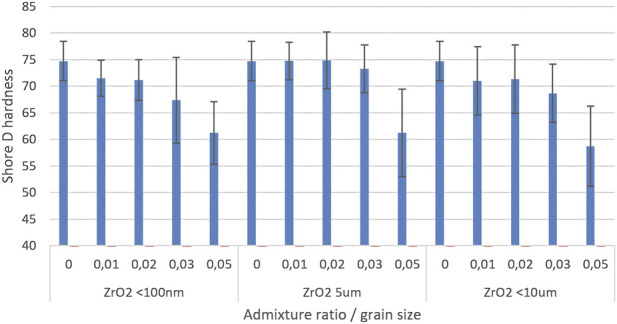
Shore D hardness (mean ± SD) of ZrO_2_-modified cements (0–5 wt%). Error bars: SD; increasing dispersion at higher concentrations.

Tests on the surface hardness of modified cements yielded clear results. It was demonstrated that the addition of 5% admixture, regardless of its granulation, leads to a noticeable softening of the samples (Figure 3). However, in the range up to 3%, no significant changes in hardness were observed compared to the reference sample, which defines a safe modification range—possible without compromising this property of the material.

For each granulation, maximum hardness occurs at the lowest additives (1%–2%), while higher concentrations result in a decrease in hardness and an increase in the dispersion of results. This phenomenon is confirmed by increasing error bars, especially when the additive content is increased, which suggests the need for a more detailed statistical analysis to confirm the observed trends. The finest fraction (<100 nm) shows the greatest susceptibility to deterioration of parameters at higher additives, but at low concentrations it provides good, repeatable results. Medium and large grain fractions (5 µm and <10 µm) do not adversely affect the hardness of cement, i.e., at low additive levels, their values are similar to those of the control samples.

The results of the final statistical analysis show that at low additions (up to 2%–3%), the admixture, regardless of granulation, does not significantly reduce the hardness of cement–the results of these samples almost always belong to one homogeneous group with the reference (Table 4). A break and decrease in hardness was confirmed when the admixture exceeded 3%, which consistently classifies the 3% and 5% samples (especially 5%) as a group significantly different from the others. The greatest reduction in hardness occurs for all fractions at a concentration of 5%, regardless of particle size. The decrease in average hardness reaches 18%–21% of the initial hardness (unmodified samples).

**TABLE 4:** Tukey’s HSD for Shore D hardness (mean ± SD; n ≥ five to six per sample). Homogeneous groups (p < 0.05).

Admixture ratio	Mean Shore D hardness	1	2	3
<100 nm				
0%	74.733	****	​	​
1%	71.525	****	****	​
2%	71.150	****	****	​
3%	67.344	​	****	****
5%	61.210	​	​	****
5 μm				
0%	74.733	****	​	​
1%	74.775	****	​	​
2%	74.878	****	​	​
3%	73.256	****	​	​
5%	61.238	​	****	​
<10 μm				
0%	74.733	****	​	​
1%	70.989	****	​	​
2%	71.330	****	​	​
3%	68.656	****	​	​
5%	58.727	​	****	​

It is important to note that when comparing different ZrO_2_ granulations at a constant additive concentration, no statistically significant differences were found between the same amount of different additives, regardless of whether it was a fraction of <100 nm, 5 μm or <10 μm fraction, the average Shore D hardness values in such comparisons form consistent homogeneous groups. This applies to each tested level: 1%, 2%, 3% and 5%. Thus, from a statistical point of view, with the same amounts of additive, differences resulting only from granulation are not significant in the context of the hardness of modified cements. This finding strongly supports the conclusion that for practical applications in this area of analysis, the safer and more decisive parameter for hardness is the amount of additive rather than its granulation.

However, for compressive strength, the amount of admixture is crucial, but at certain levels (2%, 3%), granulation can further differentiate the results–its impact is particularly important when we want to maximise this parameter. In practice, therefore, if design requirements place greater emphasis on compressive strength than on surface hardness, it is worth considering not only the concentration but also the granulation of the additive, especially at concentrations close to the optimum (2%, 3%). The optimal effect is therefore achieved at low ZrO_2_ concentrations regardless of granulation. Adding more admixture leads to both a decrease in hardness and an increase in the instability of results, which should be taken into account when designing modified cements.

## 4 Discussion

The results obtained clearly indicate that a suitably selected admixture of ZrO_2_ particles can be introduced into PMMA cement without significantly impairing its mechanical properties. All composites developed with the addition of ZrO_2_, even at a maximum concentration of 5% by weight, met the minimum compressive strength criterion according to ISO 5833 (>70 MPa). The average compressive strength values ranged from 75 to 85 MPa, similar to the control cement, which means that the structural integrity of the material was maintained. A slight decrease in strength was observed only for the highest ZrO_2_ content of 5% when larger particles (∼5 µm and <10 µm) were used, but even in these cases, the material retained the required load-bearing capacity. The finest admixture of nanometric ZrO_2_ did not adversely affect the strength of the cement; even with a 5% addition, no significant decrease in compressive strength was observed compared to unmodified cement. This means that nano-ZrO_2_ can be added in larger quantities than coarser particles without the risk of weakening the material under static load.

In contrast to compressive strength, the changes in Young’s modulus remained within a relatively narrow range and were strongly affected by data scatter, so only few pairwise comparisons reached statistical significance, indicating that the stiffening effect of ZrO_2_ is moderate and close to the measurement variability for this parameter.

Hardness analysis (Shore D) and microstructure observations allow us to understand the reasons for the observed differences. It was found that small amounts of ZrO_2_ admixtures (up to approx. 2%, 3% by weight) – regardless of particle size–do not significantly affect the hardness of hardened cement compared to unmodified cement. For these lower concentrations, the hardness values remained at a level similar to that of the control sample, indicating no negative effect on the microstructure. However, at higher additive contents, a decrease in material hardness was observed. Exceeding the threshold of ∼3% by weight of ZrO_2_ resulted in a statistically significant decrease in hardness, with the largest decrease recorded for the highest concentration of 5% (this applied to all tested ZrO_2_ fractions). Importantly, a comparison of different ZrO_2_ granulations at the same concentrations did not show significant differences in the hardness of the composites. In other words, at a given weight fraction, the effect of particle size on hardness was found to be statistically insignificant. The decrease in hardness is therefore mainly quantitative in nature and may be due to an excess of the solid phase introduced rather than the particle size itself. From a practical point of view, this means that in order to maintain the hardness and consistency of the cement structure, a certain critical level of admixture should not be exceeded (in this case, approx. 3%–5% by weight, depending on the granulation).

A change in the ZrO_2_ content within the range of one to five wt% significantly modifies the overall mechanical performance of PMMA cement, with a clearly defined structural optimum. At low additive concentrations (one to three wt%), all modified cements met the requirements of ISO 5833, and their compressive strength and Young’s modulus remained stable or improved slightly, particularly for 5 and 10 μm particles, indicating favourable stress transfer and no microstructural degradation. Only at the highest tested ZrO_2_ content (5 wt%) was a marked deterioration in mechanical properties observed, manifested as a decrease in compressive strength and a reduction in Shore D hardness by approximately 18%–21%, which can be attributed to particle agglomeration and the formation of microstructural defects. Nanoparticles (100 nm) proved to be the most compatible form of additive–even at 5 wt%, no significant decrease in static strength was observed compared to the reference cement, suggesting that they can be used at higher concentrations than coarse particles. From a mechanistic point of view, the optimal doping ratio results from a balance between uniform dispersion and the avoidance of particle clustering. At low concentrations, ZrO_2_ particles are evenly dispersed within the PMMA matrix without visible agglomerates, and their high hardness and strength (in contrast to, for example, soft BaSO_4_) increase the stiffness of the matrix, enabling more efficient load transfer and leading to higher Young’s modulus values (up to ∼1,550 MPa) and compressive strength in the upper range of 75–85 MPa for 5–10 μm particles. Good adhesion at the PMMA–ZrO_2_ interface prevents microcrack initiation, which is consistent with SEM observations showing no pronounced defects. Above 3 wt% (especially at 5 wt% for larger particles), agglomeration occurs and local clusters are formed; these agglomerates act as stress concentrators, generating micropores and structural discontinuities, which reduces strength (by more than 10 MPa), Shore D hardness (by approximately 18%–21%) and Young’s modulus (down to ∼1,200 MPa). Statistical analysis (Tukey’s HSD, p < 0.05) confirms homogeneous groups for ≤3 wt% compared to the control, but significant differences at 5 wt% addition. Larger particles (5–10 μm) at two to three wt% provide the most pronounced reinforcement (higher homogeneous groups in Table 2), due to effective crack-bridging and stable dispersion, whereas nanoparticles (100 nm) offer more stable but less pronounced stiffening (smaller increase in modulus) despite their good dispersion, which may be related to different load-sharing mechanisms operating at the nanoscale.

**FIGURE 4:**
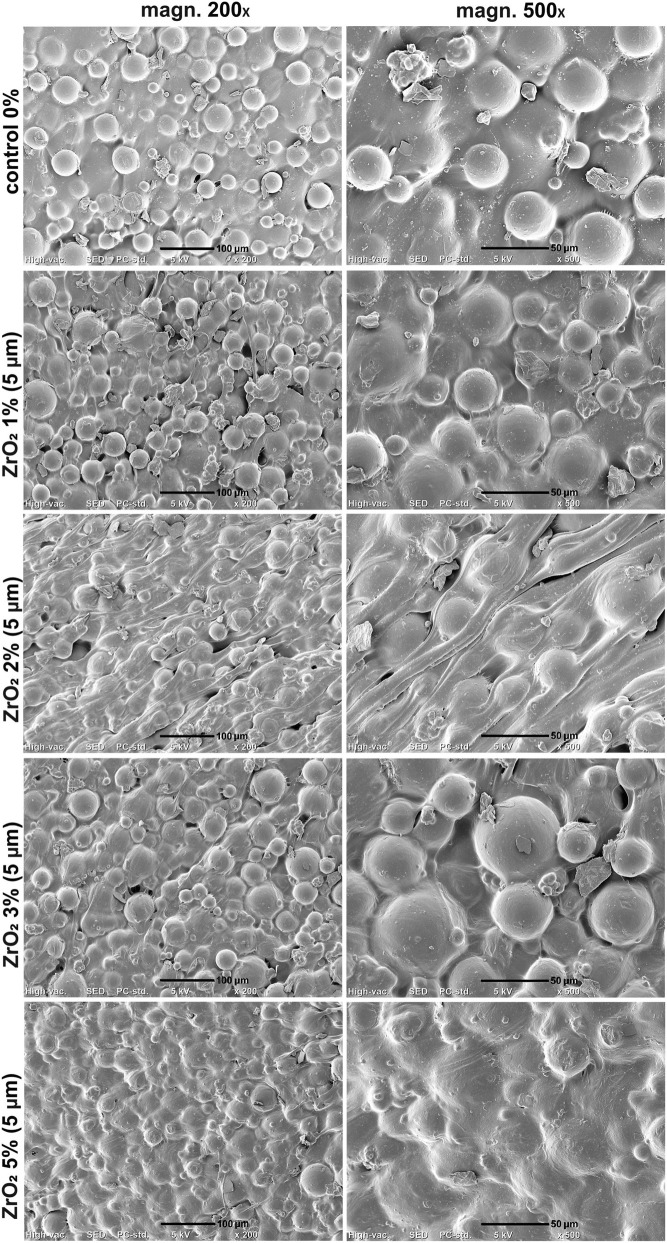
SEM micrographs of PMMA-ZrO_2_ (100 nm; 0–5 wt%) at ×200 and ×500 magnification. Scale bar: 100 μm (at 200x), 50 μm (at 500x).

**FIGURE 5:**
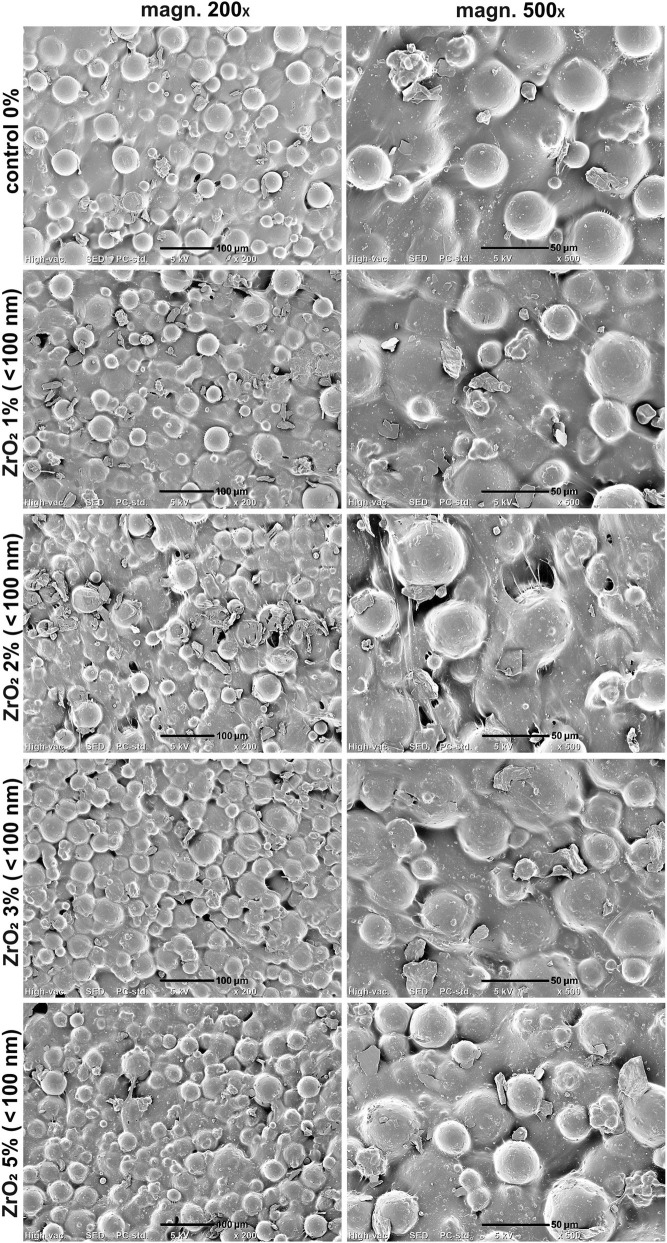
SEM micrographs of PMMA-ZrO_2_ (5 μm; 0–5 wt%) at ×200 and ×500 magnification. Scale bar: 100 μm (at 200x), 50 μm (at 500x).

**FIGURE 6:**
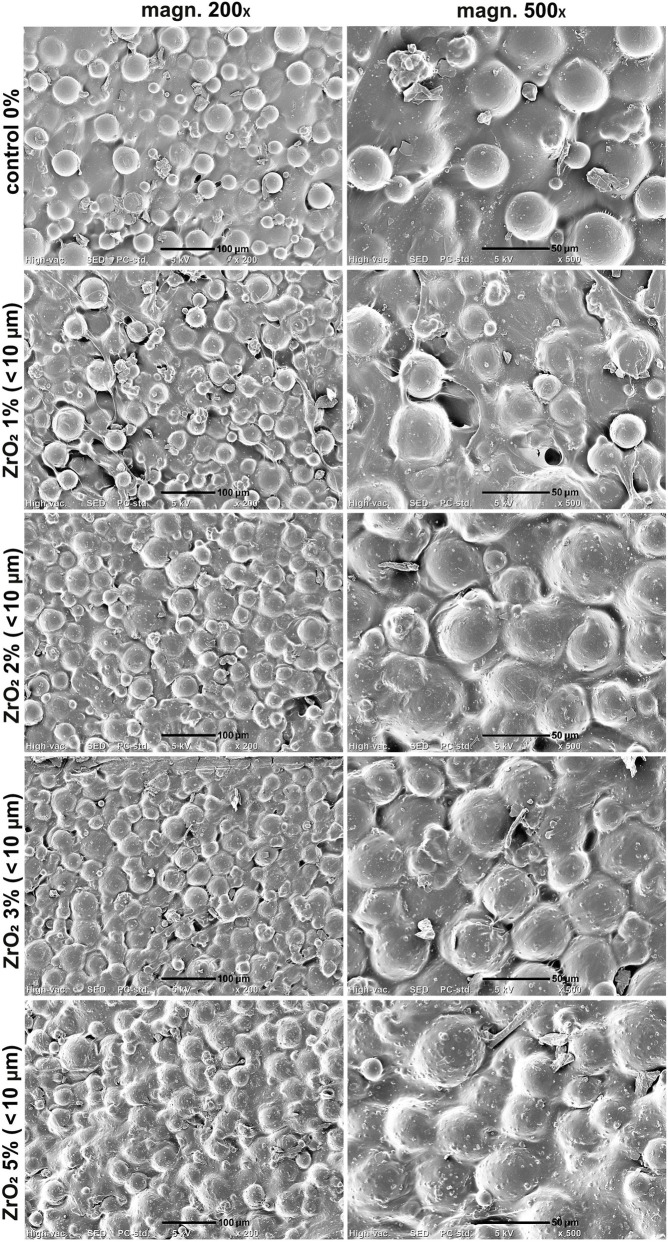
SEM micrographs of PMMA-ZrO_2_ (10 μm; 0–5 wt%) at ×200 and ×500 magnification. Scale bar: 100 μm (at 200x), 50 μm (at 500x).

SEM imaging of the samples proved uniform distribution of the PMMA beads within cement matrix (Figures 4–6). Smaller ZrO_2_ grains (<100 nm and 5 µm) were barely visible in the obtained images at applied magnifications (Figures 4, 5). ZrO_2_ particles with the size of <10 µm were uniformly distributed within material microstructure (Figure 6). Importantly, SEM analysis clearly showed that the addition of ZrO_2_ to the bone cement did not affect its microstructural properties regardless of granulation and concentration of ceramic phase. There were no evident cracks or voids that could influence mechanical properties of the bone cement. All ZrO_2_-loaded cement revealed comparable surface morphology to unmodified control sample (control 0% on the figures).

The results obtained are consistent with observations known from the literature, while also supplementing them. Previous studies suggested that too many added ceramic particles may weaken acrylic cement - Ayre et al. demonstrated a reduction in the flexural strength and fracture resistance of cement after replacing BaSO_4_ with more than 10% by weight of ZrO_2_ or TiO_2_ particles, which was explained by the agglomeration of these particles and the formation of microcracks. Our study confirms this trend, although a noticeable decrease in mechanical properties was already observed at a lower proportion (5% by weight) in the case of larger ZrO_2_ particles (5–10 µm). This is probably due to the fact that the critical concentration of the filler depends on its size and homogeneity of dispersion; large grains can generate defects at a lower volume fraction than nanoparticles. On the other hand, Chen et al. reported that the addition of nanometric ZrO_2_ up to ∼7% by weight does not impair the flexural strength of cement and even slightly increases its modulus of elasticity. In our experiment, the addition of nanoparticles (up to 5% by weight relative to unmodified cement) did not weaken the compressive strength of the material, which is consistent with these results. It can therefore be concluded that, with moderate concentrations of nanoadditives of a few percent, the strength properties of PMMA cement remain at an acceptable level or comparable to those of unmodified cement. The literature also indicates that BaSO_4_, normally present in an amount of ∼10–15% by weight, can act as a weakening inclusion, initiating fatigue cracks in the cement structure. Replacing part of this admixture with an alternative filler with greater hardness and better mechanical compatibility is therefore desirable. The selection of ZrO_2_ as a modifier of PMMA bone cement was motivated by its unique combination of high mechanical strength, chemical stability, radiopacity and proven biocompatibility, which enables simultaneous reinforcement of the polymer matrix and improvement of imaging properties without introducing cytotoxic effects. Furthermore, ZrO_2_ offers a promising alternative to conventional BaSO_4_ radiopacifiers, potentially reducing crack initiation sites while enhancing mechanical performance and biofunctional response of the composite. ZrO_2_ appears to meet these criteria–as an additive with very high hardness and strength, it can potentially increase the resistance of cement to wear and cracking, and our results show that, unlike BaSO_4_, it does not cause a significant reduction in strength at moderate concentrations. It is worth noting that the amounts of ZrO_2_ tested in this study (max. 5%) are lower than the typical BaSO_4_ content in clinical cements, and yet ZrO_2_ is known to provide adequate radiopacity even at such low concentrations (zirconium dioxide is highly contrasting). This paves the way for the development of cements with a reduced content of potentially harmful BaSO_4_, partially replaced by ZrO_2_ particles without loss of visibility on X-ray images. It has been reported in the literature that ZrO_2_ fillers can significantly accelerate PMMA polymerization: for example, Ayre et al. demonstrated that the addition of 10 wt% ZrO_2_ reduced the setting time of PMMA cement from approximately 615 s (control cement) to ∼441 s. This effect is explained by the specific interaction of the ZrO_2_ surface with the orientation of polymer chains and the course of radical polymerization (Ayre et al., 2021).

Compared to other bone cement modifiers, such as hydroxyapatite (HA) or titanium (IV) oxide (TiO_2_), the addition of zirconium dioxide appears promising. Hydroxyapatite is a well-known bioactive filler capable of forming bonds with bone, but the larger amounts of HA required for this often lead to a deterioration in the mechanical parameters of the cement due to its lower strength and lack of contrasting properties (Karpiński et al., 2024b). TiO_2_, on the other hand, has been studied mainly as an alternative opacifier–it exhibits chemical inertness similar to ZrO_2_, but with slightly lower density and hardness (Ma et al., 2011; Kim, 2020). Literature reports suggest that the effect of TiO_2_ on the mechanical properties of cement also depends on the dose and dispersion of particles, and an excessive amount (∼10% and above) may result in a reduction in strength, as in the case of ZrO_2_ [18]. Our research did not directly consider TiO_2_ or HA, but the results obtained indicate certain universal patterns: in order to improve the properties of cement without compromising its strength, it is necessary to limit the solid phase content to a level at which no structural defects arise. Zirconium dioxide seems to have the advantage that even a small addition (of the order of a few per cent) can beneficially modify the cement, providing potential reinforcement, whereas a similar osteoconductive effect would require much larger amounts of HA. Furthermore, ZrO_2_ is characterised by excellent biocompatibility and bioinertness, confirmed by long-term clinical use in other medical applications (e.g., endoprosthesis heads, dental crowns) (Gautam et al., 2016; Kunrath et al., 2021; Beus et al., 2025). Its particles do not degrade in the body environment, do not release harmful products and do not cause inflammatory reactions. Although ZrO_2_ itself is not a bioactive material in the sense of forming an apatite layer on the surface, there are indications that it may indirectly improve the biofunctionality of cement. Gillani et al. observed increased adhesion of bone cells to the surface of cement containing ZrO_2_ nanoparticles compared to pure cement and cement with BaSO_4_. This suggests that the addition of zirconium promotes colonisation of the material by cells and thus may facilitate its integration with the surrounding bone (Gillani et al., 2010). In combination with the results of the presented studies, which show no negative impact of ZrO_2_ on strength properties, this makes zirconium modification an attractive strategy for improving the biofunctionality of acrylic cements. It can be expected that PMMA cement enriched with a small amount of ZrO_2_ will not only be mechanically resistant, but also potentially more bone-friendly than conventional cements containing only BaSO_4_.

To provide a broader context for the obtained results, Table 5 presents a summary comparison of key mechanical and functional properties of PMMA-based bone cements modified with various inorganic fillers, as reported in the literature to date. The comparison encompasses a range of additive types, including ZrO_2_, TiO_2_, TaC, WC, BiS_2_, ZrO_2_ fibres, and natural fillers, and covers compressive strength, Young’s modulus, radiopacity, and selected biological characteristics where available. The data confirm that the compressive strength values obtained in the present study (75–85 MPa at optimal ZrO_2_ content of two to three wt%) are fully consistent with those reported for clinically used PMMA cements and competitive modifications, while the ZrO_2_ additive content required to achieve adequate radiopacity (≤5 wt%) is significantly lower than the standard BaSO_4_ content (10–15 wt%). Notably, while some alternative fillers, such as TaC or WC, achieve higher absolute compressive strength values (>90–100 MPa), these are attained at considerably higher filler concentrations (10–20 wt%) and involve materials of higher cost and less established long-term biocompatibility. The present system achieves a favourable balance between mechanical performance, radiopacity, low filler content, and biocompatibility, which is highlighted in Table 5.

**TABLE 5:** Comparison of mechanical and functional properties of PMMA-based bone cements modified with selected inorganic fillers - summary of literature data in relation to the present study.

Source (author/year)	Type of additive (type, concentration)	Key mechanical parameters	Radiopaque properties	Base cement	Advantages	Limitations
Gillani et al. (2010)	ZrO_2_ (micro-/nano, with and without TMS, ∼25% by weight); BaSO_4_ (micro/nano, ∼25% by weight)	Tensile: strength ∼9.3–13.0 MPa (10^6^ Pa) depending on fraction (plain 13.0), Young’s modulus ∼1.9–6.7 × 10^8^ Pa	All modifications resulted in greater radiopacity than the base cement. Additionally, nano-functionalised particles change the failure mode to ductile	PMMA (e.g., Palacos)	Increased osteoblast proliferation (higher cell density). Change in fracture mode to ductile (improved tensile strength with silanisation). High radiopacity	Reduced tensile strength (particularly without silane). Formation of microdefects (porosity) at high concentrations. Functionalisation required (silane)
Ayre et al. (2021)	TiO_2_ (anatase, silanised/unsilanised, 5%–25% by weight); t-ZrO_2_ (YSZ, 5%–25%)	Not specified. Overall, a reduction in flexural strength and modulus of elasticity (without silane) and a reduction in fracture resistance were observed with the addition of >10% TiO_2_/ZrO_2_	Cements with >10% TiO_2_/ZrO_2_ had radiopacity comparable to commercial cement with 10% BaSO_4_. Silane treatment increases the scattering and radiopacity of the material	PMMA	Alternative to BaSO_4_/ZrO_2_: biologically inert Ti/Zr. Additional bioactivity (hydroxyapatite) with silanated TiO_2_	Decrease in strength and modulus (without silane). Particle agglomeration (defects) requires a high content (>10%). Silanisation treatment required to achieve full benefits
Kotha et al. (2009)	ZrO_2_ fibres (Ø15 µm – 2% vol., Ø30 µm – 5% vol., without coating and with acrylic)	Increase in Young’s modulus and tensile strength; fracture toughness +23% (Ø15 µm, 2% vol.) and +41% (Ø30 µm, 5% vol.).	ZrO_2_ fibres provide radiopacity ≥ that of traditional additives (Zr has a high Z-). Details are not provided, but cement containing ZrO_2_ is clearly visible on X-rays	PMMA	Significant mechanical reinforcement (strength and modulus). A marked increase in fracture toughness	Volume restriction (2%–5%), as further increases may impair consistency. Potential for difficulty in uniformly mixing the fibres into the paste
Xu et al. (2024)	TaC (tantalum carbide, nanoparticles; 20% by weight in the example)	Compressive strength over 100 MPa (exceeding cement with 30% BaSO_4_). Young’s modulus increased with TaC content	Radiopacity: at 20% TaC, equivalent to the radiopacity of 30% BaSO_4_. Radiologically, the cement is highly contrastive	PMMA	Very high strength (>100 MPa). Radiopacity ≥ that of traditional cements (20% TaC ≈30% BaSO_4_). Improved osteogenesis and full biocompatibility	High cost of tantalum. High metal load (potential limitation at very high concentrations)
Xu et al. (2026)	Bi_2_S_3_ (bismuth disulphide, nanoparticles; 20% by weight)	Compressive strength 82.4 ± 3.1 MPa (>70 MPa). Young’s modulus ∼ no significant change	Radiopacity ≥ cements with 30% BaSO_4_; X-ray image clearer than with BaSO_4_	PMMA	Good strength (≥standards). Excellent biocompatibility (≥95% viable cells; trace release of Bi^3+^). Strong osteogenic effect (↑ALP 2.3×)	High weight percentage (20%). Need for monitoring of long-term Bi ion release (although low concentrations have been demonstrated)
Xu et al. (2023)	WC (tungsten carbide, nanoparticles; 10% by weight)	Compressive strength ≈90 MPa at 10% WC (≥ clinical standard). Young’s modulus ∼ unchanged	The radiopacity of cement with 10% WC is higher than in cements with 30% BaSO_4_. The cement is highly contrasted on X-rays	PMMA	High strength (∼90 MPa). Radiopacity > standard 30% BaSO_4_ cement. Lower cytotoxicity (WC is stable and insoluble)	Addition of a heavy metal (W) – cost and weight. Potential problems with nanoparticle dispersion
Antonijevic et al. (2014)	White PC (MTA) + Bi_2_O_3_ (10%–30% by weight), ZrO_2_ (10%–30%) or YbF_3_ (10%–30%)	PC with 10% Bi_2_O_3_ reduces strength (p < 0.05); 10%–30% ZrO_2_/YbF_3_ has no significant effect on compressive strength. Porosity increased significantly for all additives (p < 0.05)	A thickness of ≥3 mm Al (standard) was achieved with ≥10% Bi_2_O_3_, ≥20% ZrO_2_/YbF_3_. The radiopacity value is given in Al equivalent on the X-ray film	Portland (white), MTA	An alternative to Bi_2_O_3_: ZrO_2_/YbF_3_ achieves the required radiopacity without compromising strength. A comparison of various radiopaque agents in a single study	All additives increase porosity (weakening of the structure). Bi_2_O_3_ prolongs setting time; reduces strength, dental/endodontic cement
Liu et al. (2020)	CPC (starch-reinforced) + BaSO_4_ (percentage not specified, e.g., ‘CSB system’)	Better strength compared to CPC without additives	Radiopacity increased significantly after the addition of BaSO_4_ (the required contrast for vertebroplasty procedures was achieved)	CPC (calcium phosphate cement + starch)	Meets radiopacity requirements for vertebroplasty; increased strength and biocompatibility. Good expandability (source: clinical study in progress)	Exact values not provided (open-access article: descriptive only). Presence of starch, hybrid formulation (requires further testing)
Tavakoli et al. (2024)	Graphene oxide-emulsified baghdadite (GOBgh, nanofibres); 20% w/w (with 2% antibiotic)	Compression: +33.6% vs. Simplex® P (∼80→107 MPa); Young’s modulus +70.9%. Additionally, setting time and other properties were improved	Radiopacity improved (no precise data available, reported *as ‘increased’*). GOBgh20 cement was clearly visible on X-rays due to the heavy Zr and W atoms in baghdadite	PMMA (Simplex® P)	Significant mechanical reinforcement (↑ 33% in compression, ↑70% in modulus). Bioactive properties: improved bone integration, antibacterial functionality. Industrially interesting combination of graphene + ceramics	High loading (20%). Complex, multi-component formulation (may present manufacturing difficulties). No comprehensive data on long-term stability and fatigue strength
This research	ZrO_2_ (particles of varying particle size, 0%–5% by weight)	Compressive strengths decrease at >3% ZrO_2_; optimally, up to 3% does not significantly reduce strength	Not analysed	PMMA (Palamed)	The possibility of improving X-ray contrast with a small addition of ZrO_2_ was confirmed. Positive biophysical and chemical effects (surface apatitisation) were observed	Exceeding the optimum (approx. 3%) weakens the cement (decrease in strength). Further testing required (fatigue, ageing)

The concentration-dependent reinforcing effect of inorganic fillers is, however, not unique to PMMA-based systems and has been consistently documented across a broad range of polymer matrices. For instance, nano α-Al_2_O_3_ particles at an optimum content of 4 wt% increase the tensile strength (∼16% above pure PP) and elastic modulus of polypropylene composites, while higher filler contents (≥5 wt%) lead to agglomeration and deterioration of those properties (Mirjalili et al., 2014). Similarly, TiO_2_ filler at an optimum of 4 wt% maximises the tensile and flexural strength of Kevlar/epoxy hybrid composites, with mechanical performance declining at 6 wt% due to particle aggregation (Natrayan et al., 2023). These findings confirm that the existence of an optimal filler concentration–beyond which structural defects outweigh the reinforcing effect–is a universal phenomenon in polymer–ceramic composites, irrespective of both the specific filler chemistry and the polymer matrix. The type and chemistry of the filler play an equally important role: in epoxy-based adhesive composites, the addition of natural fillers such as montmorillonite, chalk (CaCO_3_), or activated carbon at 2 wt% significantly modifies tensile strength and elongation at break, with the direction and magnitude of the effect depending on both the filler type and the specific resin system used (Miturska et al., 2020). Furthermore, the mechanical performance of polymer composites is governed not only by the filler content, but also by the overall formulation: in epoxy adhesive systems, the choice of curing agent alone can change shear strength by more than sixfold, confirming that all compositional parameters must be optimised simultaneously to achieve maximum mechanical performance (Miturska-Barańska et al., 2022). A similar concentration-dependent behaviour has been reported for epoxy-based composites: the compressive strength of filled epoxy adhesive systems varies significantly depending on the type and content of the inorganic filler, demonstrating that filler selection and dosage must be carefully optimised to avoid structural deterioration (Ogrodniczek et al., 2025). Among potential ceramic additives to PMMA, zirconium oxide occupies a special place. ZrO_2_ has long been used as a contrast agent in bone cements–the addition of this compound ensures good visibility of the cement on X-ray images, comparable to traditional BaSO_4_. Unlike soft barium sulphate, ZrO_2_ particles are characterised by very high hardness, strength and chemical stability. As a biocompatible, non-reactive ceramic material, ZrO_2_ has been proposed as a reinforcing filler that can improve the mechanical properties of cement without adversely affecting the polymerisation process. Literature reports indicate that small additions of ZrO_2_ nanoparticles can have a beneficial effect on the strength of hardened cement - e.g., Chen et al. observed that modifying cement with ∼7% by weight of ZrO_2_ (from nanoparticles coated with an antibacterial layer) did not reduce the strength tested in a flexural test, and even increased its modulus of elasticity by 3%–14% compared to unmodified cement. Furthermore, zirconium oxide is characterised by excellent biocompatibility, confirmed by many years of use of this material in medical implants. Its particles do not dissolve or degrade in a physiological environment, thus they do not introduce harmful products into the surrounding tissues. It has even been noted that the addition of fine-grained ZrO_2_ may promote interaction between the material and bone tissue–*in vitro* studies have shown significantly greater adhesion of osteoblast cells to the surface of cement modified with ZrO_2_ nanoparticles than to pure cement or cement containing only conventional BaSO_4_. This improvement in cellular response suggests that the addition of zirconium may increase the biofunctionality of the cement, potentially facilitating its integration with living bone.

The results of this study have direct practical implications for orthopedic biomaterial engineering. It has been shown that by adding an appropriately selected amount of ZrO_2_, it is possible to obtain bone cement with improved characteristics while maintaining critical strength parameters. From the point of view of the orthopedic surgeon and the patient, this means a potentially more durable material—more resistant to cracking and abrasion during many years of implant use. In addition, such cement provides good X-ray visibility, which facilitates monitoring the stability of the endoprosthesis after surgery. Based on the results obtained, it can be recommended that when modifying PMMA cement with zirconium dioxide, the concentration should not exceed approx. 3% by weight if medium or large particles are used, while in the case of nanoparticles, a content of up to ∼5% by weight can be considered, provided that they are uniformly dispersed. These ranges guarantee the required compressive strength and hardness, while at the same time allowing the intended benefits to be achieved (strengthening, increased wear resistance, improved biofunctionality). In clinical practice, it would make sense to partially replace traditional BaSO_4_ in the cement formulation with ZrO_2_—for example, reducing the BaSO_4_ content from 15% to 10% and adding 3%–5% ZrO_2_ could improve the fatigue resistance of the cement without compromising its performance properties. An additional advantage of such a modification is the possibility of functionalizing ZrO_2_ particles, e.g., by coating them with bioactive or antibacterial layers. PMMA cements with ZrO_2_ nanoparticles containing antibacterial compounds have already been described in the literature, which retained their mechanical parameters while exhibiting antibacterial activity (Tanvir et al., 2025). This opens up the prospect of designing multifunctional bone cements that combine high strength, radiopacity, and the ability to reduce the risk of periprosthetic infections.

The proposed modification of acrylic cement with zirconium dioxide allows for structural optimisation of the material–the appropriate selection of ZrO_2_ granulation and concentration enables the reinforcement of PMMA cement without compromising its integrity. PMMA/ZrO_2_ cements are a promising alternative to conventional single-phase bone cements, offering a combination of clinically relevant performance characteristics. In order to fully exploit their potential, further research is recommended, particularly in the areas of long-term fatigue strength and interaction with bone tissue *in vivo*. Nevertheless, the results obtained so far can serve as a basis for the development of new bone cement formulations with improved durability and biofunctionality, which in the future may translate into longer endoprosthesis life and improved patient outcomes.

The findings of this study provide a quantitative framework for optimizing the particle size and concentration of ZrO_2_ fillers in PMMA-based bone cements, which can guide the rational design of next-generation composite biomaterials with tailored mechanical and functional properties. In particular, the identification of optimal reinforcement ranges (two to three wt% for micro-scale particles and up to ∼5 wt% for nanoparticles) highlights the importance of balancing dispersion and agglomeration effects, forming a basis for future studies on multifunctional modifications, including bioactive and antibacterial functionalization. Furthermore, the demonstrated structure–property relationships create opportunities for integrating experimental results with computational modeling and long-term biological evaluation in subsequent research.

Importantly, these findings also have direct practical and industrial relevance, as they provide clear formulation guidelines for the development of commercially viable bone cements with improved durability and controlled radiopacity. The possibility of partially replacing conventional radiopacifiers (e.g., BaSO_4_) with ZrO_2_ enables the design of advanced cement systems with enhanced fatigue resistance and potential for further functionalization, supporting the translation of these composites into next-generation orthopedic biomaterials.

## 5 Limitations and future perspectives

One of the main limitations of this study is the relatively narrow scope of the mechanical tests performed. The tests mainly included static compression tests in accordance with ISO 5833 and hardness measurements, while shear stress and bending tests as well as material brittleness analyses were omitted. The lack of long-term fatigue tests makes it difficult to assess the durability of cement under cyclic loads–PMMA cements exhibit relatively low fatigue strength and brittleness, which in the long term promotes the initiation of microcracks and material damage. Future studies should therefore include fatigue (cyclic) tests and additional mechanical tests (e.g., bending or shear tests) to comprehensively assess the behaviour of composite cement under conditions similar to actual clinical loads.

Extending the tests to conditions similar to the physiological environment is also necessary. The mechanical tests were conducted under standard conditions (room temperature, dry samples), without simulating the effects of body fluids, elevated temperature or high humidity. Furthermore, technological and environmental factors, such as the presence of blood, bone fragments or saline during cement application, can reduce its mechanical parameters. In addition, the PMMA polymerisation process is exothermic, which in the presence of tissue can lead to thermal necrosis and the release of residual monomer with a cytotoxic effect. In subsequent stages of the study, it is worth incubating the experimental samples in artificial body fluid (SBF) at body temperature and assessing their mechanical properties after this procedure. This will allow us to check whether moisture accumulation or ion interaction changes the strength or stiffness of the modified cements.

A significant quantitative limitation is the range of ZrO_2_ additive concentrations. In the studies, a maximum of 5% filler by weight relative to unmodified cement was assumed. For comparison, the standard content of the radiopaque additive BaSO_4_ in clinical cements is approximately 10%–15% by weight. Limiting the study to low concentrations of ZrO_2_ means that the effect of a higher ceramic phase content on the properties of the cement was not assessed. According to the authors’ observations, the addition of 5% ZrO_2_ ensured full X-ray contrast while reducing the proportion of BaSO_4_, but the effects of replacing most of the BaSO_4_ with ZrO_2_ or adding, for example, 10%–15% by weight of ZrO_2_ have not yet been investigated. Further research is therefore needed to determine the optimal concentrations and proportions of the BaSO_4_/ZrO_2_ mixture in terms of mechanics and contrast, as well as the possible reinforcement of the cement while maintaining the required strength parameters.

The study did not include an assessment of the biological properties of the composites. No cytotoxicity tests or studies of osteoblast cell adhesion or proliferation on the modified cement surface (*in vitro* studies) were performed, nor were any *in vivo* analyses conducted. This is a significant limitation, as even biologically inert additives can affect tissue integration. The literature points to the potential biological benefits of ZrO_2_, e.g., Gillani et al. found higher osteoblast density on ceramics-containing cements, especially nanofunctionalised ones, compared to pure cement (Gillani et al., 2010). Furthermore, Hossain et al. demonstrated improved bone regeneration in a rat model using PMMA cement with HA and β-TCP additives, evaluating the effects using micro-CT and histological analysis (Hossain et al., 2023). Based on this, future studies should include a comprehensive analysis of biocompatibility: *in vitro* tests (cytotoxicity, osteoblast cell adhesion and proliferation, inflammatory response) and *in vivo* experiments. Statements regarding the biofunctional potential of PMMA/ZrO_2_ composites are based on physicochemical and microstructural characteristics as well as literature reports indicating enhanced osteoblast adhesion to ZrO_2_-modified cements. Consequently, these findings should be interpreted with caution, and further *in vitro* (cytotoxicity, osteoblast adhesion) and *in vivo* studies are required to validate the biological performance of the developed materials. Only a combination of ZrO_2_ cement modification.

Another important direction is the use of numerical biomechanical models. Previous work has focused on laboratory measurements without simulating the actual loads on the bone-cement-implant system. In the future, it would be worthwhile to develop FEM models based on tissue data to predict the distribution of stresses and deformations in reinforced cement during use. For example, Mu et al. combined clinical analyses with FEM models to study cemented spinal screw fixation (Mu et al., 2025). Such simulations would complement experimental data and help optimise the modification design, e.g., by assessing how different ZrO_2_ concentrations affect load transfer in the bone structure.

Future studies will include comprehensive *in vitro* mineralization assays, such as simulated body fluid (SBF) immersion, to evaluate the bioactivity and apatite-forming ability of PMMA composites modified with varying ZrO_2_ fractions, thereby enabling a detailed assessment of the influence of filler characteristics on mineralization behavior. Additionally, long-term incubation of PMMA/ZrO_2_ composite cements in SBF will be conducted to investigate potential changes in mechanical performance and microstructural stability under physiologically relevant conditions, which is essential for assessing their long-term clinical durability. To further elucidate the physicochemical interactions between the PMMA matrix and the zirconium oxide filler, advanced characterization techniques, including Fourier-transform infrared spectroscopy (FTIR) and X-ray diffraction (XRD), will be employed to identify interfacial interactions, phase composition, and structural modifications induced by ZrO_2_ incorporation.

In summary, further research should include: extended mechanical testing (especially long-term fatigue loading and new failure modes), 3D microstructure analysis, testing under conditions simulating the biological environment (type of fluids and temperature), as well as comprehensive *in vitro* and *in vivo* biological evaluations and numerical modelling. Only such a comprehensive scope of research will allow for full verification of the potential of PMMA-ZrO_2_ cement composites and optimisation of their formulations in terms of interaction with bone tissue.

## 6 Conclusion

In the tested PMMA-based bone cements with zirconium oxide (ZrO_2_) additive, a significant influence of particle size and additive concentration on their mechanical properties was demonstrated. An increase in the proportion of fine ZrO_2_ particles led to an increase in compressive strength, Young’s modulus and material hardness. The highest compressive strength values were achieved for the smallest grain fractions at a moderate concentration of ZrO_2_. Too high a concentration of the additive or the use of excessively large particles resulted in the formation of aggregates and the appearance of micropores in the composite structure, which led to a reduction in mechanical parameters.

Microstructural analyses revealed that the homogeneous dispersion of fine ZrO_2_ particles in the PMMA matrix promotes effective stress transfer and reduces structural defects. Good adhesion between the ceramic particles and the polymer matrix resulted in increased stiffness and strength of the cement. On the other hand, the presence of large ZrO_2_ agglomerates generated local stress concentrations and reduced load-bearing capacity, confirming a strong correlation between microstructure and mechanical test results.

Based on the test results, the range of optimal parameters for the ZrO_2_ additive was determined. The most favourable reinforcement was obtained with fine particles (several hundred nanometres in size) and moderate concentration (several per cent by weight). In this range, the properties of the cement improved significantly without compromising the cohesion of the matrix. Exceeding the optimal concentration value, on the other hand, caused densification of the ceramic phase, which reduced the effectiveness of the reinforcement and impaired the homogeneity of the microstructure.

The results confirmed that ZrO_2_ can act as both a reinforcing and radiopaque filler in PMMA cements. Due to its high density and atomic number, zirconium oxide increases the radiopacity of the cement, which allows it to be clearly visualised in X-ray examinations. The significance of these findings lies in the possibility of designing new bone cements with optimised mechanical and radiological properties. The indicated combination of grain size and additive concentration provides important guidelines for the design of composite materials for orthopaedic applications.

## Data Availability

The raw data supporting the conclusions of this article will be made available by the authors, without undue reservation.

## References

[B1] Amerstorfer F. Fischerauer S. Sadoghi P. Schwantzer G. Kuehn K. D. Leithner A. (2017). Superficial vancomycin coating of bone cement in orthopedic revision surgery: a safe technique to enhance local antibiotic concentrations. J. Arthroplasty 32, 1618–1624. 10.1016/j.arth.2016.11.042 28111125

[B2] Antonijevic D. Medigovic I. Zrilic M. Jokic B. Vukovic Z. Todorovic L. (2014). The influence of different radiopacifying agents on the radiopacity, compressive strength, setting time, and porosity of Portland cement. Clin. Oral Invest. 18, 1597–1604. 10.1007/s00784-013-1130-0 24233183

[B3] Ayre W. N. Scully N. Elford C. Evans B. A. Rowe W. Rowlands J. (2021). Alternative radiopacifiers for polymethyl methacrylate bone cements: silane-treated anatase titanium dioxide and yttria-stabilised zirconium dioxide. J. Biomater. Appl. 35, 1235–1252. 10.1177/0885328220983797 33573445 PMC8058833

[B4] Beus J. H. W. D. Cune M. S. Meijer H. J. A. Raghoebar G. M. Schepke U. (2025). Metal‐free custom‐made zirconia implants—A prospective 5‐Year follow‐up single‐arm clinical trial. Clin. Implant Dent. Rel Res. 27, e13404. 10.1111/cid.13404 39506212 PMC11789843

[B5] Buchholz T. Siverino C. Moriarty T. F. Sheehy E. J. O’Brien F. J. Nehrbass D. (2025). Antibiotic‐loaded polymer‐calcium phosphate scaffold for treating orthopedic device‐related infection in a rabbit segmental bone defect model. J. Biomed. Mater. Res. 113, e37917. 10.1002/jbm.a.37917 40296342

[B6] Cen C. Zhang Y. Cao Y. Hu C. Tang L. Liu C. (2025). Construction of a 3D degradable PLLA/β-TCP/CS scaffold for establishing an induced membrane inspired by the modified single-stage masquelet technique. ACS Biomater. Sci. Eng. 11, 1629–1645. 10.1021/acsbiomaterials.4c01849 39943835 PMC11900768

[B7] Chen Y. Caneli G. Almousa R. Xie D. (2022). A novel antibacterial zirconia-containing PMMA bone cement. J. Mech. Behav. Biomed. Mater. 129, 105135. 10.1016/j.jmbbm.2022.105135 35279449

[B8] Constant C. Stroncek J. D. Zeiter S. Arens D. Nehrbass D. Gehweiler D. (2022). Venous injection of a triphasic calcium-based implant in a sheep model of pulmonary embolism demonstrates minimal acute systemic effects. Eur. Spine J. 31, 2812–2821. 10.1007/s00586-022-07303-x 35976438

[B9] Enache A.-V. Toader C. Onciul R. Costin H. P. Glavan L.-A. Covache-Busuioc R.-A. (2025). Surgical stabilization of the spine: a clinical review of spinal fractures, spondylolisthesis, and instrumentation methods. JCM 14, 1124. 10.3390/jcm14041124 40004655 PMC11856911

[B10] Frank F. A. Krampitz B. Steiner J. Strathausen R. Morgenstern M. Clauss M. (2025). Evaluation and testing of polymethylmetacrylic (PMMA) bone cements with admixed amphotericin B. J. Orthop. Surg. Res. 20, 151. 10.1186/s13018-025-05565-x 39920816 PMC11806800

[B11] Gautam C. Joyner J. Gautam A. Rao J. Vajtai R. (2016). Zirconia based dental ceramics: structure, mechanical properties, biocompatibility and applications. Dalton Trans. 45, 19194–19215. 10.1039/C6DT03484E 27892564

[B12] Ghasemi F. Jahani A. Moradi A. Ebrahimzadeh M. H. Jirofti N. (2023). Different modification methods of poly methyl methacrylate (PMMA) bone cement for orthopedic surgery applications. ABJS 11, 485–492. 10.22038/abjs.2023.71289.3330 37674694 PMC10479821

[B13] Gillani R. Ercan B. Qiao A. Webster T. J. (2010). Nanofunctionalized zirconia and barium sulfate particles as bone cement additives. Int. J. Nanomedicine 5, 1–11. 10.2147/IJN.S7603 20161983 PMC2819907

[B14] Gürses A. Ejder-Korucu M. (2016). “Poly (methyl Methacrylate)(PMMA),” in CAE DS-Inject. Mould. Mater, 6501–6510.

[B15] Hong L. Hjort K. Andersson D. I. Persson C. (2025). Linoleic acid addition prevents *Staphylococcus aureus* biofilm formation on PMMA bone cement. Biofilm 10, 100311. 10.1016/j.bioflm.2025.100311 40823343 PMC12357060

[B16] Hossain M. Jeong J. H. Sultana T. Kim J. H. Moon J. E. Im S. (2023). A composite of polymethylmethacrylate, hydroxyapatite, and β‐tricalcium phosphate for bone regeneration in an osteoporotic rat model. J. Biomed. Mater. Res. 111, 1813–1823. 10.1002/jbm.b.35287 37289178

[B17] Humez M. Citak M. Luck S. Linke P. Gehrke T. Paul C. (2025). Enhancing PMMA cements with manually added antimicrobial agents. APMIS 133, e70029. 10.1111/apm.70029 40411307

[B18] Hussain S. Al-Sarraf A. (2022). Influence of bioactive and bio inert ceramic powders on tribology properties of PMMA composite denture base. JBBBE 57, 1–8. 10.4028/p-3f74k7

[B19] Karpiński R. Szabelski J. (2025). Mechanical properties and functional assessment of PMMA bone cements modified with glassy carbon. JFB 16, 254. 10.3390/jfb16070254 40710468 PMC12295586

[B20] Karpiński R. Szabelski J. Maksymiuk J. (2019a). Effect of physiological fluids contamination on selected mechanical properties of acrylate bone cement. Materials 12, 3963. 10.3390/ma12233963 31795371 PMC6926979

[B21] Karpiński R. Szabelski J. Maksymiuk J. (2019b). Seasoning polymethyl methacrylate (PMMA) bone cements with incorrect mix ratio. Materials 12, 3073. 10.3390/ma12193073 31547178 PMC6804204

[B22] Karpiński R. Szabelski J. Krakowski P. Jonak J. Falkowicz K. Jojczuk M. (2024a). Effect of various admixtures on selected mechanical properties of medium viscosity bone cements: part 1 – α/β tricalcium phosphate (TCP). Compos. Struct. 343, 118306. 10.1016/j.compstruct.2024.118306

[B23] Karpiński R. Szabelski J. Krakowski P. Jonak J. Falkowicz K. Jojczuk M. (2024b). Effect of various admixtures on selected mechanical properties of medium viscosity bone cements: part 2 – hydroxyapatite. Compos. Struct. 343, 118308. 10.1016/j.compstruct.2024.118308

[B24] Karpiński R. Szabelski J. Krakowski P. Jonak J. Falkowicz K. Jojczuk M. (2024c). Effect of various admixtures on selected mechanical properties of medium viscosity bone cements: part 3 – glassy carbon. Compos. Struct. 343, 118307. 10.1016/j.compstruct.2024.118307

[B25] Karpiński R. Prus A. Krakowski P. Paśnikowska-Łukaszuk M. Jonak K. (2025). Diagnostic approaches to total knee arthroplasty loosening: from conventional imaging to modern techniques. Appl. Sci. 16, 445. 10.3390/app16010445

[B26] Kim H.-K. (2020). Optical and mechanical properties of highly translucent dental zirconia. Materials 13, 3395. 10.3390/ma13153395 32751942 PMC7435650

[B27] Kim M. J. Park S. Y. Kang S. Lee Y. J. Lee S.-J. Kim J. T. (2025). Low modulus PMMA-Based bone cement for the reduction of adjacent vertebral fractures after vertebroplasty. Acta Biomater. 203, 399–411. 10.1016/j.actbio.2025.07.053 40714190

[B28] Kırkbınar M. İbrahimoğlu E. Yetgin S. H. Çalışkan F. (2024). Investigation of tribological behavior of poly(methyl methacrylate) biocomposite containing hydroxyapatite. Emerg. Mater. Res. 13, 15–28. 10.1680/jemmr.23.00039

[B29] Kotha S. Li C. Schmid S. Mason J. (2009). Reinforcement of bone cement using zirconia fibers with and without acrylic coating. J. Biomed. Mater. Res. 88A, 898–906. 10.1002/jbm.a.31783 18384160

[B30] Krishnan M. R. Alshabib R. M. Alsharaeh E. H. (2025a). Effect of silver/reduced graphene oxide@titanium dioxide (Ag/rGO@TiO2) nanocomposites on the mechanical characteristics and biocompatibility of poly(Styrene-co-methyl Methacrylate)-Based bone cement. Polymers 17, 1970. 10.3390/polym17141970 40732849 PMC12300378

[B31] Krishnan M. R. Alsoughayer S. Michael F. M. Alsharaeh E. H. (2025b). Poly(styrene-*co* -methyl methacrylate)-silver/reduced graphene oxide-nano hydroxyapatite nanocomposites for bone cement applications. Int. J. Polym. Mater. Polym. Biomaterials 74, 657–668. 10.1080/00914037.2024.2372791

[B32] Kuehn K.-D. Ege W. Gopp U. (2005). Acrylic bone cements: composition and properties. Orthop. Clin. N. Am. 36, 17–28. 10.1016/j.ocl.2004.06.010 15542119

[B33] Kumar A. (2025). Biomechanics of interfaces for cemented joint replacements: a systematic review. Next Mater. 9, 100943. 10.1016/j.nxmate.2025.100943

[B34] Kumar A. Ghosh R. (2021). Fracture toughness of acrylic PMMA bone cement: a mini-review. IJOO 55, 1208–1214. 10.1007/s43465-021-00495-2 34824722 PMC8586281

[B35] Kunrath M. F. Gupta S. Lorusso F. Scarano A. Noumbissi S. (2021). Oral tissue interactions and cellular response to zirconia implant-prosthetic components: a critical review. Materials 14, 2825. 10.3390/ma14112825 34070589 PMC8198172

[B36] Kwong J. W. Abramowicz M. Kühn K. D. Foelsch C. Hansen E. N. (2024). High and low dosage of vancomycin in polymethylmethacrylate cements: efficacy and mechanical properties. Antibiotics 13, 818. 10.3390/antibiotics13090818 39334991 PMC11428212

[B37] Liu H. Zhang Z. Gao C. Bai Y. Liu B. Wang W. (2020). Enhancing effects of radiopaque agent BaSO4 on mechanical and biocompatibility properties of injectable calcium phosphate composite cement. Mater. Sci. Eng. C 116, 110904. 10.1016/j.msec.2020.110904 32806278

[B38] Liu Z. Wen L. Zhou L. Liu Z. Chen Y. Geng B. (2024). Comparison of cemented and cementless fixation in total knee arthroplasty: a meta-analysis and systematic review of RCTs. J. Orthop. Surg. Hong. Kong 32, 10225536241267270. 10.1177/10225536241267270 39564945

[B39] Ma J. F. Wang S. Q. Du R. X. Li X. D. (2011). Pressureless sintering of gelcast ZTA–MgO–TiO_2_ systems as potential dental ceramics. Adv. Appl. Ceram. 110, 275–279. 10.1179/1743676111Y.0000000010

[B40] Mashak A. Atai M. Nodehi A. (2025). The impact of different radiopacifying agents on the elution and mechanical properties of vancomycin-loaded bone cements. AAPS PharmSciTech 26, 222. 10.1208/s12249-025-03221-5 40866736

[B41] Mirjalili F. Chuah L. Salahi E. (2014). Mechanical and morphological properties of polypropylene/nano *α* -Al_2_ O_3_ composites. Sci. World J. 2014, 1–12. 10.1155/2014/718765 24688421 PMC3932228

[B42] Miturska I. Rudawska A. Müller M. Valášek P. (2020). The influence of modification with natural fillers on the mechanical properties of epoxy adhesive compositions after storage time. Materials 13, 291. 10.3390/ma13020291 31936413 PMC7013851

[B43] Miturska-Barańska I. Rudawska A. Doluk E. (2022). Influence of physical modification of the adhesive composition on the strength properties of aerospace aluminum alloy sheet adhesive joints. Materials 15, 7799. 10.3390/ma15217799 36363390 PMC9657623

[B44] Mu X. Wei X. Nong J. Ye H. Li Z. Wei M. (2025). Clinical evaluation and finite element analysis of bone cement-augmented anterolateral screw fixation *versus* percutaneous bilateral pedicle screw fixation co-applied with oblique lumbar interbody fusion for single-level lumbar degenerative diseases with osteoporosis. Front. Bioeng. Biotechnol. 13, 1571849. 10.3389/fbioe.2025.1571849 40557307 PMC12185528

[B45] Natrayan L. Janardhan G. Paramasivam P. Dhanasekaran S. (2023). Enhancing mechanical performance of TiO2 filler with kevlar/epoxy-based hybrid composites in a cryogenic environment: a statistical optimization study using RSM and ANN methods. Front. Mat. 10, 1267514. 10.3389/fmats.2023.1267514

[B46] Ogrodniczek J. Rudawska A. Müller M. (2025). Comparative analysis of compressive strength of epoxy adhesive compounds containing fillers. J. Adhesion 101, 331–355. 10.1080/00218464.2023.2286295

[B47] Santos M. A. Cardoso M. J. B. Farias K. A. S. Santos K. O. Morúa O. C. Fook M. V. L. (2019). Influência da incorporação da HAp e β-TCP no cimento ósseo wollastonita/brushita. Matéria (Rio J.) 24, e12398. 10.1590/s1517-707620190003.0711

[B48] Sari W. R. Fauzan R. Kalijaga M. H. A. Arjasa O. P. Prajatelistia E. (2024). The effect of pH and temperature on the nano zirconium oxide synthesis to enhance the radiopacity of PMMA bone cement. Coimbatore, India. 10.1063/5.0242819

[B49] Seesala V. S. Sheikh L. Basu B. Mukherjee S. (2024). Mechanical and bioactive properties of PMMA bone cement: a review. ACS Biomater. Sci. Eng. 10, 5939–5959. 10.1021/acsbiomaterials.4c00779 39240690

[B50] Szoradi G. T. Feier A. M. Zuh S. G. Russu O. M. Pop T. S. (2024). Polymethyl methacrylate bone cement polymerization induced thermal necrosis at the cement–bone interface: a narrative review. Appl. Sci. 14, 11651. 10.3390/app142411651

[B51] Tanvir M. A. H. Khaleque M. A. Kim G.-H. Park S.-E. Lee H.-H. Kim Y.-Y. (2025). Low-temperature spine-specific PMMA enhances bone regeneration *via* localized thermal necrosis in an osteoporotic rat model. IJMS 26, 4786. 10.3390/ijms26104786 40429929 PMC12112048

[B52] Tavakoli M. Najafinezhad A. Mirhaj M. Karbasi S. Varshosaz J. Al-Musawi M. H. (2024). Graphene oxide-encapsulated baghdadite nanocomposite improved physical, mechanical, and biological properties of a vancomycin-loaded PMMA bone cement. J. Biomaterials Sci. Polym. Ed. 35, 823–850. 10.1080/09205063.2024.2308328 38300323

[B53] Wilczyński M. Bieniek M. Krakowski P. Karpiński R. (2024). Cemented vs. cementless fixation in primary knee replacement: a narrative review. Materials 17, 1136. 10.3390/ma17051136 38473607 PMC10933953

[B54] Xu T.-G. Liu D.-C. Wang Y. Chen S. Li B. Zhang F. (2023). Tungsten carbide-enhanced radiopaque and biocompatible PMMA bone cement and its application in vertebroplasty. Compos. Commun. 40, 101615. 10.1016/j.coco.2023.101615

[B55] Xu T.-G. Shi J. Qi H. Chen S. Li B. Zhang F. (2024). Radiopaque and biocompatible PMMA bone cement triggered by nano tantalum carbide and its osteogenic performance. ACS Biomater. Sci. Eng. 10, 5624–5631. 10.1021/acsbiomaterials.4c00552 39107258

[B56] Xu T.-G. Gao L.-X. Liu Y. Chen D. Zhang F. He J.-H. (2026). Bismuth chalcogenides: multifunctional enhancement of radiopacity, mechanical resilience, and osteogenesis in PMMA bone cements for vertebroplasty. J. Mat. Chem. B 14, 995–1002. 10.1039/D5TB02051D 41431793

[B57] Zhou Y. Höglund L. Samanta A. Procter P. Persson C. (2024). Hydroxyapatite particle shape affects screw attachment in cancellous bone when augmented with hydroxyapatite-containing hydrogels. J. Mech. Behav. Biomed. Mater. 150, 106241. 10.1016/j.jmbbm.2023.106241 37995601

